# Is adolescent internet use a risk factor for the development of depression symptoms or vice-versa?

**DOI:** 10.1017/S0033291723000284

**Published:** 2023-10

**Authors:** Caroline Fitzpatrick, Annie Lemieux, Jonathan Smith, Greg L. West, Véronique Bohbot, Mark Asbridge

**Affiliations:** 1 Department of Preschool and Elementary School Education, University of Sherbrooke, Sherbrooke, Canada; 2 Department of Childhood Education, University of Johannesburg, Johannesburg, South Africa; 3 Department of Psychology, University of Montreal, Montreal, Canada; 4 Department of Psychiatry, McGill University, Montreal, Canada; 5 Department of Community Health and Epidemiology, Dalhousie University, Halifax, Canada

**Keywords:** Adolescents, depression, digital media, internet use, longitudinal modeling

## Abstract

**Background:**

The extent to which digital media use by adolescents contributes to poor mental health, or vice-versa, remains unclear. The purpose of the present study is to clarify the strength and direction of associations between adolescent internet use and the development of depression symptoms using a longitudinal modeling approach. We also examine whether associations differ for boys and girls.

**Methods:**

Data are drawn from (*N* = 1547) participants followed for the Quebec longitudinal Study of Child Development (QLSCD 1998–2020). Youth self-reported internet use in terms of the average hours of use per week at the ages of 13, 15, and 17. Youth also self-reported depression symptoms at the same ages.

**Results:**

After testing sex-invariance, random intercepts cross-lagged panel models stratified by sex, revealed that internet use by girls was associated with significant within-person (time-varying) change in depression symptoms. Girl's internet use at age 13 was associated with increased depression symptoms at age 15 (ß = 0.12) and internet use at age 15 increased depression at age 17 (ß = 0.10). For boys, internet use was not associated with significant time varying change in depression symptoms.

**Conclusions:**

The present findings support the hypothesis that internet use by adolescents can represent a significant risk factor for the development of depressive symptoms, particularly in girls.

The World Health Organization projects that major depression will be a leading cause of disease burden by 2030 (Bucksch et al., 2016). Adolescent's experience of depressive symptoms predicts later risk of developing major depressive disorder and impairment into adulthood (Jones, 2013; Naicker, Galambos, Zeng, Senthilselvan, & Colman, 2013; Pine, Cohen, Cohen, & Brook, 1999). Furthermore, the experience of poor mental health and depression during adolescence is linked to academic difficulties, interpersonal challenges, and decreased quality of life and earning potential (Naicker et al., 2013). The experience of depressive symptoms can lead youth to self-medicate, resulting in the adoption of unhealthy habits such as smoking, increased alcohol consumption, and illicit drugs use (Harris & Edlund, 2005). Over the span of the last decade, depression and suicide rates have climbed significantly among youth, particularly for girls (Hedegaard, Curtin, & Warner, 2021; Keyes, Gary, O'Malley, Hamilton, & Schulenberg, 2019; Lin et al, 2016). This trend towards worse mental health has raised concerns that the rising popularity of digital media use among youth may be to blame for their worsening mental health (Twenge, Joiner, Rogers, & Martin, 2018).

In a recent survey, 45% of youth reported being online ‘almost constantly’ (Pew Research Center, 2018). Research has linked digital media use by adolescents to increased depression symptoms cross-sectionally (Lin et al., 2016; Twenge et al., 2018). However, cross-sectional studies are unable to establish a clear direction of effects. In addition, most studies have been unable to rule out the possibility that adolescents with more depression symptoms may choose to spend more time online. For this reason, some have disputed the existence of a causal and clinically meaningful relationship between media use and adolescent depression (Lin et al., 2016). One exception to these methodological limits is Boers, Afzali, Newton, and Conrod (2019), who provided longitudinal evidence that digital media use by adolescents is a causal risk factor for the development of depression symptoms in Canadian youth. To inform public health interventions, replications with independent samples are required. Furthermore, Boers et al., did not address the possibility that associations between time spent online and depression symptoms may differ for boys and girls. Previous studies have reported sex-based differences in the effect of digital media use on youth depression symptoms and have found sex differences in the etiology and prevalence rates of mood disorders (Ophir, Lipshits-Braziler, & Rosenberg, 2020). Furthermore, boys and girls are likely to have different experiences online, as girls spend more time engaged in social and interactive forms of internet use, while boys are more likely to engage in gaming and view adult online websites (Ciarrochi et al., 2016). As such, it remains important to address whether the association between internet use and depression is the same in boys and girls.

Our aim is to better understand the direction of associations between adolescent internet use and depressive symptoms in a Canadian population-based sample. Given the prior literature, we hypothesize that higher levels of internet use will be associated with inter-individual increases in symptoms of depression over time. We also examine the extent to which patterns of associations differ for boys and girls and predict that associations will be stronger for girls.

## Methods

### Sample

The present study draws on data collected between 2011 and 2015 when youth were 13, 15, and 17 years old, in the context of the Quebec longitudinal Study of Child Development (QLSCD 1998–2018). The QLSCD was planned and implemented by the *Institut de la Statistique du Québec*. The sample originates from a randomly selected, stratified sample of 2837 infants born between 1997 and 1998 in the province of Quebec, Canada. At the onset of the study, 49.1% of participants were girls, 72% were described by their parent or primary caregiver as being Canadian, and 21.7% of parents reported being under the poverty line cut-off for Canadian families. The third phase of this study, upon which our research draws, was conducted to better understand the psychosocial and academic adjustment of youth during high school.

In total, data from 1547 participants (*n* = 897 girls and *n* = 740 boys) were analyzed. Participants were retained in the analytic sample if they provided data at one of the data collection waves at ages 13, 15, or 17. In terms of the distribution of missing data for boy and girls, respectively, 11.6 and 9.0% had data available at only one measurement wave, 29.1 and 21.2% had data at two measurement waves, and finally 59.3 and 69.8% had available data at all three waves. Little's test was computed to evaluate if data were missing completely at Random (MCAR). This test was non-significant for both girls and boys (χ^2^ = 9.987, df = 9, *p* = 0.352; χ^2^ = 8.839, df = 9, *p* = 0.452), indicating that missing data was not systemic. We used full-information maximum likelihood estimation to handle missing data in our analyses.

### Procedure

Questionnaires were administered predominantly in French, reflecting the linguistic distribution of the province of Quebec, Canada. This study received approval from the ethics review boards of the *Quebec Institute of Statistics*. From school entry onward, informed consent was obtained from the child and parents. The main predictors for this study were collected when participants were in their first, third, and fifth year of high school, respectively.

### Predictors

Screen time. At ages 13, 15, and 17 youth self-reported how much time they spent per week accessing the internet on a computer to play browser games, do searches, chat or go on Facebook (excluding time spent on the internet at school). Questionnaires are available here (www.jesuisjeserai.stat.gouv.qc.ca/informations_chercheurs/outils_collecte/outils_collecte_an.html). Response options included: (1) None; (2) Less than an hour; (3) 1 to 2 h; (4) 3 to 5 h; (5) 6 to 10 h; (6) 11 to 14 h; (7) 15 to 20 h; or (8) more than 20 h. Scores were converted to measures of hours per week by using the midpoint value for each range except for 20 or more hours which was scored as 20 h.

### Outcome measures

Depression age 13. At age 13 youth self-reported depression symptoms over the past two weeks based on their agreement with eight statements such as I hate myself, or I do most things well (inverse item), Cronbach's alpha = 0.79. These items were developed by the Quebec Institute of Statistics for the purpose of the *Quebec Longitudinal Study of Child Development*. All scores were converted to a mean ranging from 0 to 10, with higher scores reflecting higher levels of depression.

Depression age 15 and 17. In 2013 and 2015, the QLSCD included a special mental health and adjustment component. Youth reported depression symptoms over the past 12 months using an online questionnaire which has been previously validated (Sidani, Shensa, Hoffman, Hanmer, & Primack, 2016). Youth responded to eight items (i.e. I felt I wasn't as good-looking or as smart as other people, Nothing was fun for me, I wasn't interested in anything) scored on a Likert scale from 1 (never true) to 3 (always true), Cronbach's alpha = 0.84. Scores were converted to means ranging from 0 to 10, with 10 indicating the highest levels of symptomology.

### Data analysis strategy

Our aim is to examine whether a pattern of temporal associations exists between adolescent internet use and depression symptoms and whether associations are the same for boys and girls. We begin by testing sex-invariance for the variances and covariances of all study variables. More specifically, we assess if the variance/covariance matrix structure is the same for boys and girls. The χ^2^ improvement test is considered between the model freely estimated across groups and then compared to the fixed model. A significant test would indicate that the variance/covariance matrix differs enough to reject the hypothesis of equal structures among boys and girls.

To verify the longitudinal relationship between average weekly hours spent surfing the internet and depression symptoms, we employ a random-intercept cross-lagged panel model (RI-CLPM). Our model was estimated across the ages of 13, 15 and 17 (13Y, 15Y and 17Y). The RI-CLPM model proposed by Hamaker, Kuiper, and Grasman (2015) extends the auto-regressive cross-lagged model (CLPM) by splitting variance into between- (stable) and within- (time varying) person components. The stable component is represented by two latent random intercepts loading on internet use and depression symptoms, respectively, across all time-points. The time varying component is captured by a latent factor at each wave which represents changes from one's own mean level of depression symptom as a function of changes in one's own level of internet use. As was the case with CLPM, autoregressive effects of depression and internet use are assessed from the previous assessment point of the same measure and cross-lagged effects across measures are assessed from two consecutive time point. The model was tested via structural equation modeling (SEM) with *Mplus* 8.6 (Muthén & Muthén, 2020) using the robust full-information maximum likelihood estimator. Model fit was assessed following the benchmarks proposed by Hu and Bentler (1999) for global fit measures (Root Mean Square Error of Approximation, RMSEA; Comparative Fit Index, CFI; Chi-Square test of model fit, χ^2^). Hu and Bentler suggest a RMSEA < 0.05 and a CFI > 0.95 as a conservative criterion for good model fit. As for the value of χ^2^, it should ideally be small enough not to reach the significance level (*p* > 0.05).

An advantage of this modeling approach is that it helps address residual confounding by partitioning the variance in the repeated outcome measure (depression symptoms) into variance of interest (time-invariant, dynamic within person) from the stable, between-person component. Simulation studies have indicated that this approach can reduce the occurrence of spurious associations and bias in directional estimates of association, and more closely approximates causal inference than multivariable regression or standard cross-lagged panel models (Berry & Willoughby, 2017; Hamaker et al., 2015). For these reasons we employ this analytical strategy in favor of standard cross-lagged panel models or multiple regression models adjusted for confounders. A similar analytical approach has been used to investigate the association between child digital media use and the emergence of developmental delays (Madigan, Browne, Racine, Mori, & Tough, 2019).

## Results

### Descriptive statistics and correlations

Descriptive statistics are presented in Table 1 for boys and girls separately. Girls reported significantly higher depression scores at all ages. Girls also reported more internet use than boys at 15Y. However, boys and girls did not differ in the amount of time spent using the internet at the ages of 13Y and 17Y. Finally, as shown in Table 2, mean hours of internet use increased significantly between 13Y and 15Y for both boys and girl and only for boys between 15Y and 17Y. Mean depression scores also increased between 15Y and 17Y for both boys and girls (13Y not the same scale). Bivariate correlations at the between-subject level, stratified by sex, are presented in Table 3. Depression scores and internet use showed developmental continuity for both boys and girls. Furthermore, internet use and depression symptoms were consistently correlated for both boys and girls between the ages of 13 and 17, though this pattern was stronger and more consistent for girls.

**Table 1.** Descriptive statistics for study variables stratified by sex

	Boys mean (s.d.)	Girls mean (s.d.)	*p* value
Depression (13Y)	1.40 (1.54)	1.71 (1.69)	0.001
Depression (15Y)	2.64 (1.95)	4.27 (2.22)	<0.0001
Depression (17Y)	3.01 (2.10)	4.49 (2.28)	<0.0001
Internet use (13Y)	5.35 (5.78)	5.48 (5.44)	0.708
Internet use (15Y)	6.67 (6.06)	7.34 (5.97)	0.039
Internet use (17Y)	7.82 (6.51)	7.31 (6.00)	0.162

*Note.* Internet use is measured in hours/week. Data were compiled from the final master file of the Québec Longitudinal Study of Child Development (2011–2015), ^©^Gouvernement du Québec, Institut de la statistique du Québec.

**Table 2.** Paired samples *t* tests stratified by sex indicating change in depression symptoms and internet use between the ages of 13 and 17

	Boys		Girls	
	*p* value	Cohen d	*p* value	Cohen d
Depression (13Y–15Y)	–	–	–	–
Depression (15Y–17Y)	<0.0001	0.21	0.029	0.09
Internet use (13Y–15Y)	<0.0001	0.17	<0.0001	0.33
Internet use (15Y–17Y)	0.010	0.12	0.205	0.05

*Note.* Depression (13Y–15Y) not applicable. Data were compiled from the final master file of the Québec Longitudinal Study of Child Development (2011-2015), ^©^Gouvernement du Québec, Institut de la statistique du Québec.

**Table 3.** Correlations between adolescent internet use and depression symptoms stratified by sex

	1	2	3	4	5	6
Boys						
1. Depression (13Y)	–	0.34***	0.33***	0.08	0.02	0.14**
2. Depression (15Y)		–	0.52***	0.15**	0.18***	0.02
3. Depression (17Y)			–	0.13**	0.14**	0.15**
4. Internet use (13Y)				–	0.35***	0.25***
5. Internet use (15Y)					–	0.36***
6. Internet use (17Y)						–
Girls						
1. Depression (13Y)	–	0.39***	0.33***	0.16***	0.11**	0.04
2. Depression (15Y)		–	0.55***	0.17***	0.24***	0.06
3. Depression (17Y)			–	0.14**	0.21***	0.14***
4. Internet use (13Y)				–	0.35***	0.18***
5. Internet use (15Y)					–	0.32***
6. Internet use (17Y)						–

*Note.* Internet use is measured in hours/week. *** denotes alpha < 0.0001, ** denotes alpha < 0.01, and * denotes alpha < 0.05. Data were compiled from the final master file of the Québec Longitudinal Study of Child Development (2011–2015), ^©^Gouvernement du Québec, Institut de la statistique du Québec.

### Sex invariance

The χ^2^ sex-invariance test between the two models was significant: Δχ^2^ = 415, Δdf = 21, *p* = 0.0001. This indicates that relaxing the equality constraints of the variance/covariance matrix between boys and girls improved the model significantly, thus providing support for the hypothesis that these matrices are different. In addition, the means of four of the six variables were also different between boys and girls. As such, statistical, empirical, and theoretical rationales provide support for conducting sex-based analyses.

### Longitudinal Models Using RI-CLPM

Table 3 presents the results of estimated models for boys and girls separately. Both models generated very good fit indices (RMSEA < 0.05, CFI > 0.95 and nonsignificant χ^2^) [boys: (RMSEA = 0.000, CFI = 1.000 and χ^2^(1) = 0.284, *p* = 0.594; girls: RMSEA = 0.000, CFI = 1.000 and χ^2^(1) = 0.841; *p* = 0.359).

**Fig. 1.**
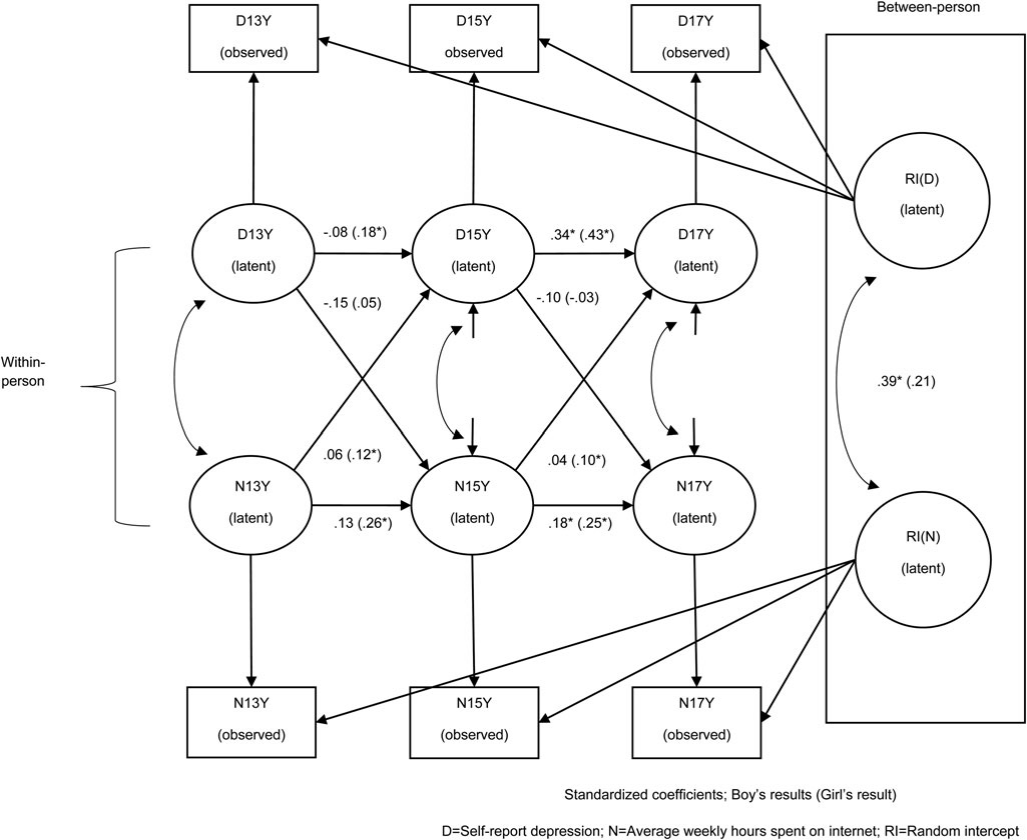
Random intercept cross-lagged panel model examining associations between internet use (*N*) and depressive symptoms (D) adolescent in boys and girls.

As shown in Fig. 1, the model estimating association between internet use and depression in boys revealed a significant covariance of intercepts at the stable (between-person) level *b* = 0.12, s.e. = 0.05, *p* = 0.012). This indicates that on average boys who spent more time online also reported more symptoms of depression across all three time points. Within person, the stable, auto-regressive path from 13Y to 15Y were non-significant, indicating that depression symptoms and internet use at 13Y did not predict later depression symptoms and internet use respectively at age 15Y. However, the auto-regressive path coefficients between 15Y and 17Y were significant indicating some stability in depression symptoms and internet use between the ages of 15 and 17, respectively (depression: *b* = 0.38, s.e. = 0.06, *p* < 0.0001; internet use: *b* = 0.19, s.e. = 0.08, *p* = 0.021).

For girls a different pattern emerged. The intercepts' covariance at the stable level (between-person) was not significant (*b* = 0.04, s.e. = 0.09, *p* = 0.144). This indicates that on average girls who spent more time using the internet across all study waves, did not have higher depression scores. For girls at the within person-level, auto-regressive associations were significant across all time points indicating relative rank order stability over time in internet use and depression at the ages of 13, 15 and 17 (depression 13Y–15Y: *b* = 0.26, s.e. = 0.12, *p* = 0.033; depression 15Y–17Y: *b* = 0.44, s.e. = 0.05, *p* < 0.0001; internet use 13Y–15Y: *b* = 0.30, s.e. = 0.08, *p* < 0.0001; internet use 15Y–17Y: *b* = 0.25, s.e. = 0.07, *p* < 0.0001). Finally, the cross-lagged path coefficients indicate that the more hours girls spent using the internet at 13 and 15, the more symptoms of depression they reported at age 15 and 17 (13Y–15Y: *b* = 0.47, s.e. = 0.19, *p* = 0.013; 15Y–17Y: *b* = 0.35, s.e. = 0.15, *p* = 0.017) at the time varying level. These time-varying associations at the within person-level statistically controlled for stable, between-person differences (Table 4).

**Table 4.** RI-CLPM longitudinal relationship between major depression symptoms and average weekly hours spent on the internet for boys and girls separately

	Boys (*n* = 740) χ^2^ = 0.284, df = 1, *p* value = 0.594; RMSEA = 0.000; CFI = 1.000			Girl (*n* = 807) χ^2^ = 0.841, df = 1, *p* value = 0.359; RMSEA = 0.000; CFI = 1.000		
	*b* (s.e.)	*p* value	Beta *ß*	*b* (s.e.)	*p* value	Beta *ß*
Between-person covariance						
RI(*N*) ↔ RI(*D*)	0.12 (0.05)	0.01	0.39	0.04 (0.05)	0.441	0.21
Within-person regression paths						
N13Y → N15Y	0.14 (0.09)	0.144	0.13	0.30 (0.08)	0.000	0.26
D13Y → N15Y	−0.07 (0.05)	0.113	−0.15	0.02 (0.02)	0.304	0.05
N15Y → N17Y	0.19 (0.08)	0.021	0.18	0.25 (0.07)	0.000	0.25
D15Y → N17Y	−0.04 (0.02)	0.085	−0.10	−0.01 (0.01)	0.570	−0.03
D13Y → D15Y	−0.12 (0.20)	0.546	−0.08	0.26 (0.12)	0.033	0.18
N13Y → D15Y	0.20 (0.28)	0.473	0.06	0.47 (0.19)	0.013	0.12
D15Y → D17Y	0.38 (0.06)	0.000	0.34	0.44 (0.05)	0.000	0.43
N15Y → D17Y	0.13 (0.19)	0.518	0.04	0.35 (0.15)	0.017	0.10

*Note*. *b*, unstandardized regression coefficient; *ß*, standardized regression coefficient; *D*, Depression symptoms; *N*, Internet use (hours/week); RI, Random intercept. Note. Data were compiled from the final master file of the Québec Longitudinal Study of Child Development (2011–2015), ^©^Gouvernement du Québec, Institut de la statistique du Québec.

### Clinical significance

Effect sizes were small to moderate. More specifically, our analyses suggest that an increase of one standard deviation in girls internet use (approximately 50 min per day), corresponds to a 0.12 and 0.10% standard deviation increases in depression scores two and four years later.

## Discussion

In the present study, we examined associations between adolescent internet use and emerging depression symptoms, separately for boys and girls. Our analytic strategy allowed us to specifically estimate the contribution of within-individual changes in internet use on subsequent increases in depressive symptoms while also considering whether depression symptoms contribute to internet use. Our results indicated that internet use was consistently associated with inter-individual increases in depression symptoms for girls in a manner that suggests that internet use may be a significant risk factor for the development of depression symptoms. For boys, associations were observed at the between-group level only. An inverse association, whereby more symptoms of depression contribute to increases in media consumption, was not born out in our data. Effect sizes for the present study were in the low to moderate range and were comparable to those observed in other studies (Boers et al., 2019; Ciarrochi et al., 2016).

The results of previous longitudinal studies examining associations between adolescent screen media use and internalizing mental health problems have provided a mixed picture. Some have found evidence for bidirectionality between screen use and later depression (Houghton et al., 2018). Others have found small, negative associations between screen media use and adolescent wellbeing (Orben & Przybylski, 2019). These diverging results could be explained by the heterogeneity in methodological approaches used across studies. For instance, the present study examined depression symptoms as an outcome, whereas others have examined mental wellbeing or life satisfaction (Orben & Przybylski, 2019; Orben, Dienlin, & Przybylski, 2019). Furthermore, many studies have combined boys and girls in their analyses, which can obscure sex and gender differences in sensitivity to screen use. A recent review finds that associations are strongest between adolescent screen use and depression, as opposed to other internalizing problems or more general measures of wellbeing (Tang, Werner-Seidler, Torok, Mackinnon, & Christensen, 2021). Furthermore, according to the same review, the directionality of associations matters, with studies providing stronger effects sizes between screen use and depression than vise versa. Finally, the use of newer forms of screen media (ex., surfing the internet, smart phone use) by youth appears to be more strongly associated with risk of depression than older forms of screen use (ex., television viewing).

Some studies suggest that internet use may benefit youth mental health under the right circumstances. For instance, youth often report that time spent online leads them to feel less lonely and more confident (Rideout & Robb, 2018). Other research indicates that time spent online may allow youth to feel more connected to others and to help them maintain their existing friendships (Bessière, Kiesler, Kraut, & Boneva, 2008; Valkenburg & Peter, 2009). Being able to identify online activities and content that are most damaging, along with those that are beneficial, can be useful for establishing harm-reduction approaches to inform adolescent media use interventions. Furthermore, a better understanding of the circumstances under which individual vulnerabilities and strengths may give rise to positive and negative outcomes remains an important area of investigation.

There are several reasons internet use could lead youth to develop depression symptoms. First, youth who spend much of their time interacting with screen media are likely to experience reduced opportunities for face-to-face interactions with peers. According to a recent study, based on a large representative sample of American youth, there has been a generational decrease in the amount of time adolescents spend engaging in face-face interactions (Twenge, Spitzberg, & Campbell, 2019). In particular, the amount of time youth spent getting together with friends has decreased by an average of 1 h a day in current generations.

We observed negative individual time-varying effects for girls but not boys. This may reflect sex- and gender-based differences in adolescents' on-line experiences. Although boys and girls spend similar amounts of time online, boys and girls differ with respect to their media diets. First, girls are more likely to use the internet to access social media sites (Jurewicz, 2015). Social media use has been linked to increased symptoms of depression and anxiety, and decreased self-esteem, emotional wellbeing, and life satisfaction (Boers et al., 2019; Ophir et al., 2020; Seedat et al., 2009). Each of these in turn represent important psychological risk factors for the development of mood disorders. This may be the case because social media platforms can promote negative social comparisons with other users that may seem to be happier and more successful (Lup, Trub, & Rosenthal, 2015).

Girls are also more likely to be exposed to objectifying images and to engage in negative appearance-related social comparisons than boys (Ciarrochi et al., 2016; Myers & Crowther, 2009). Research has indicated that women report more negative feelings after upward social comparisons than men (Fox & Vendemia, 2016). More time spent online may therefore increase girls' exposure to contents that lead to repeated upward social comparisons which can be detrimental to self-esteem and mental health (Seedat et al., 2009). Previous research has also shown that adolescent girls report lower self-esteem than boys, particularly for appearance-related dimensions of self-esteem (Gentile, Grabe, Dolan-Pascoe, Twenge, & Wells, 2009). As such, it may be the case that girls are more vulnerable to certain types of internet use, such as social media engagement.

Social media use is also thought to influence reward prediction error. Tonic dopamine release in areas of the basal ganglia increases when unpredicted rewards occur, but not when predicted rewards occur. Importantly, when predicted rewards do not occur, individuals experience a *decrease* in dopamine release, resulting in symptoms of withdrawal (Schultz, 1998). Social media interactions (e.g. receiving a like on a post or a positive comment; engaging in social comparisons) are likely to contribute to the experience of unexpected rewards or the absence of expected rewards. The latter could then cause psychological withdrawal contributing to depressive symptoms. Withdrawal symptoms, in turn, may lead to reward seeking behavior expressed with increased internet use, thereby completing a negative feedback loop that could lead to further dopamine depletion. Indeed, dopamine dysregulation is associated with symptoms of depression in adolescents (Forbes & Dahl, 2012).

A strength of the present study was our ability to establish the direction of effects between internet use and depressive symptoms in adolescents. As such, we can shed light on what comes first, poor mental health or internet use. Furthermore, it was possible to examine developmental associations over four years spanning between the ages of 13 and 17. Finally, we were able to observe associations in a large population-based sample and to describe how patterns of associations may differ for boys and girls.

In terms of limitations, our correlational design prevents us from ruling out the possibility that associations are due to common risk factors such as genetic predisposition or environmental influences. Another limitation is our use of data collected between 2011 and 2015. As such, it is possible that the nature of youth online activities have shifted since, given the rising popularity of apps such as Tik Tok, Instagram, and SnapChat. Similarly, it was not possible to account for the specific types activities youth were engaged with while online. For instance, it remains important to better understand which online activities (i.e. messaging, social media use, gaming) are most closely linked to mental health outcomes in boys and girls.

As a modifiable risk factor, youth media habits may represent an important intervention target. There is evidence that parental monitoring of child media habits including setting limits, and actively discussing contents can lead to improvements in academic performance and interpersonal behavior (Cox et al., 2009). As a result, health professionals can play an important role by encouraging parents and youth themselves to establish family plans and strategies for supporting healthy screen use habits.

The potential harms and benefits of media use by adolescents remains an important preoccupation. Furthermore, news outlets frequently report seemingly contradictory findings which has led to the public perception that there is scant evidence to support a link between media use and mental health. Our results suggest that internet use by adolescent girls may represent a significant environmental risk factor for the development of depression symptoms.

## Supplementary material

For supplementary material accompanying this paper visit https://doi.org/10.1017/S0033291723000284. click here to view supplementary material
