# Supplementary material for: Is adolescent internet use a risk factor for the development of depression symptoms or vice-versa?
Source: Psychol Med. 2023 Feb 24;53(14):6773–9. doi: 10.1017/S0033291723000284 (PMC10600816; doi:10.1017/S0033291723000284)
Supplement: Supplementary file 1 [file S0033291723000284sup001.docx]

**SUPPLEMENTARY MATERIAL**

**Is adolescent internet use a risk factor for the development of depression symptoms or vice-versa?**

Fitzpatrick, C, Lemieux, A., Smith J, West, G.L. Bohbot, V, Asbridge, M.

**Mplus syntax**

usevariables are

N13Yobs N15Yobs N17Yobs

D13Yobs D15Yobs D17Yobs;

missing are all (-999);

useobs are (sex eq 2);

DEFINE:

N13Yobs=N13Yobs/10;

N15Yobs=N15Yobs/10;

N17Yobs=N17Yobs/10;

ANALYSIS:

model = nocovariances;

MODEL:

RIN by N13Yobs@1 N15Yobs@1 N17Yobs@1;

RID by D13Yobs@1 D15Yobs@1 D17Yobs@1;

N13Ylat by N13Yobs@1;

N15Ylat by N15Yobs@1;

N17Ylat by N17Yobs@1;

D13Ylat by D13Yobs@1;

D15Ylat by D15Yobs@1;

D17Ylat by D17Yobs@1;

N13Yobs-N17Yobs@0;

D13Yobs-D17Yobs@0;

N15Ylat on N13Ylat D13Ylat;

N17Ylat on N15Ylat D15Ylat;

D15Ylat on D13Ylat N13Ylat;

D17Ylat on D15Ylat N15Ylat;

RIN with RID;

N13Ylat with D13Ylat;

N15Ylat with D15Ylat;

N17Ylat with D17Ylat;

OUTPUT: sampstat stand modindices (3.84) residual tech4;
